# An Analysis of Whether Health Literacy and Numeracy Are Associated with Diabetes Medication Adherence

**DOI:** 10.3928/24748307-20171212-01

**Published:** 2018-01-23

**Authors:** Arathi S. Nandyala, Lyndsay A. Nelson, Andrea E. Lagotte, Chandra Y. Osborn

## Abstract

Many people with type 2 diabetes (T2D) do not take medications as prescribed, resulting in suboptimal glycemic control and a greater risk of diabetes complications. Taking medications regularly requires adequate health literacy and numeracy skills, but associations between health literacy and numeracy skills and medication adherence have been mixed. We used validated, reliable, and widely accepted measures to examine the relationship between health literacy, numeracy, and medication adherence among a sample of adults with T2D. We analyzed cross-sectional data using nonparametric Mann-Whitney U tests and unadjusted and adjusted logistic regression models. For every 1 point increase on the Brief Health Literacy Screen and Subjective Numeracy Scale, participants were 1.8 and 2.7 times more likely to optimally take medications (*p* < .05). Health literacy and numeracy skills should be considered in the design of education materials for diabetes medication management and adherence. **[*HLRP: Health Literacy Research and Practice*. 2018;2(1):e15–e20.]**

People with type 2 diabetes (T2D) often require oral antihyperglycemic agents and/or insulin to achieve and maintain optimal glycemic control. However, not taking diabetes medications is common. As many as one-third of people with T2D do not take their medication as prescribed (Kirkman et al., 2015). Not taking antihyperglycemic medications and/or insulin as directed has been linked to suboptimal glycemic control (Aikens & Piette, 2013), and a greater risk of hospitalizations and premature mortality (Currie et al., 2012).

Successful medication taking requires access to treatment, and, once in hand, proper self-administration. Treatment access includes, but is not limited to, affordability of prescriptions (Bailey et al., 2012; Mayberry, Mulvaney, Johnson, & Osborn, 2017) and adequate transportation to and from pharmacies (Bailey et al., 2012). Upon receipt of the prescription, a person must understand how to store it, dose it, handle a missed dose (e.g., double up or skip it), prevent and manage side effects, remember to take the medication, and refill the prescription and/or request a doctor to reauthorize it. These tasks require sufficient health literacy and numeracy skills.

Health literacy is the “degree to which individuals have the capacity to obtain, process, and understand basic health information and services needed to make appropriate health decisions” (Kindig, Panzer, & Nielsen-Bohlman, 2004), whereas numeracy is the “ability to understand and use numbers” in a health care setting (Rothman, Montori, Cherrington, & Pignone, 2008). Adequate health literacy and numeracy skills are consistently associated with having more diabetes knowledge, but associations with self-care behaviors such as medication taking and clinical outcomes (e.g., glycemic control) have been mixed (Bailey et al., 2014).

Understanding the role of health literacy and numeracy skills in taking diabetes medications may inform efforts to optimize medication adherence and, in turn, glycemic control. With cross-sectional data from a sample of people with T2D, we examined relationships between health literacy, numeracy, medication adherence, and glycemic control. Because conceptual (Paasche-Orlow & Wolf, 2007) and empirical evidence (Osborn, Paasche-Orlow, Bailey, & Wolf, 2011) suggests self-care is in the predicted pathway between health literacy/numeracy and outcomes, we did not test the relationship between literacy/numeracy and glycemic control. Instead, we examined the relationship between literacy/numeracy and medication adherence, and then, separately, the relationship between medication adherence and glycemic control.

## Methods

### Participants

We analyzed baseline, cross-sectional data collected from 151 adults with T2D recruited for a medication adherence randomized controlled trial at Vanderbilt University Medical Center (VUMC) in Nashville, TN. Eligible participants were at least age 18 years, English-speaking, diagnosed with T2D, had a glycated hemoglobin A1c (A1c) of ≥6.5% within 3 months of enrollment, and were prescribed at least one diabetes medication and/or insulin at enrollment. Exclusion criteria included severe hearing or visual impairments, delirium or a severe cognitive impairment, having a caregiver who administered diabetes medications, not having a “My Health at Vanderbilt” patient portal account, not having a mobile phone or computer with Internet access, and being unwilling or unable to provide informed consent.

### Procedures

Data collection and study procedures were approved by the Vanderbilt University Institutional Review Board. We employed a variety of recruitment strategies. We displayed flyers and recruitment cards at VUMC clinics, advertised the study on the medical center's listserv and Research Match (an online database of people interested in participating in research studies), and identified people through the Research Derivative (a program that identifies potential participants in Vanderbilt's clinical systems). A trained research assistant (RA) sent potential participants information about the study and an electronic survey to screen for eligibility. People who screened eligible were invited to review and sign an electronic informed consent document. The RA also sent them instructions for obtaining an A1c test, and a link to the REDCap^TM^ (Research Electronic Data Capture) baseline survey.

### Measures

**Demographic and diabetes characteristics.** The baseline survey collected self-reported age, gender, race, education, income, and duration of diagnosed diabetes. Education was operationalized as the highest grade completed from eight options (**Table 1**). Participants selected 1 of 8 income options, with the lowest option being less than $25,000, the highest being more than $90,000, with the other options in $15,000 increments between the lowest and highest options. Participants also reported how long they had been diagnosed with diabetes in years and months. A trained RA reviewed each participant's electronic medical record to obtain insurance status (private, public, or no insurance), insulin use, and the number of prescribed diabetes medications (both based on a participant's medication list).

**Health literacy skills.** We assessed health literacy skills with the 3-item Brief Health Literacy Screen (BHLS) (Wallston et al., 2014). The BHLS has high internal consistency reliability, inter-administrator reliability, and concurrent validity (Wallston et al., 2014). We reverse scored items as recommended and then summed scores across items to create a composite (range, 3–15). Higher scores indicated better health literacy skills, per the method used by Wallston et al. (2014).

**Numeracy skills.** We assessed numeracy skills with the 8-item Subjective Numeracy Scale (SNS) (Fagerlin et al., 2007). The SNS has concurrent validity with objective measures of numeracy, but takes less time, and is less stressful and frustrating to complete (Fagerlin et al., 2007). We reverse scored items as recommended and then averaged scores across items to create a composite (range, 1–6). Higher scores indicated better numeracy skills.

**Medication adherence.** We assessed medication adherence with the 11-item Adherence to Refills and Medications Scale for Diabetes (ARMS-D) (Mayberry, Gonzalez, Wallston, Kripalani, & Osborn, 2013). The ARMS-D has high internal consistency, and convergent validity with other self-report measures of diabetes medication adherence (Mayberry et al., 2013). Scores are summed and range from 11 to 44. We reversed scored responses, such that higher scores indicated better medication adherence. We dichotomized ARMS-D scores to reflect optimal (perfect score of 44) or suboptimal (score less than 44) medication adherence.

**Glycemic control.** A RA reviewed each participant's electronic medical record to obtain the most recent A1c test result and its associated date.

## Analyses

All statistical tests were performed using SPSS version 24.0. Descriptive statistics characterized the sample, and Mann-Whitney U tests and chi-square tests examined differences between participants who had optimal versus suboptimal adherence according to the ARMS-D. Unadjusted and adjusted logistic regression models examined the relationships between participants' health literacy skills and medication adherence, and, separately, their numeracy skills and medication adherence. We adjusted for participants' age, gender, race, education, income, insurance status, insulin use, duration of diagnosed diabetes, and the number of prescribed diabetes medications. We also used Mann-Whitney U tests to examine the relationship between medication adherence and glycemic control. Because conceptual (Paasche-Orlow & Wolf, 2007) and empirical evidence (Osborn, Paasche-Orlow, et al., 2011) suggests self-care is in the predicted pathway between health literacy and numeracy and outcomes, we did not test the relationship between health literacy and glycemic control. We did, however, use Mann-Whitney U tests to examine the relationship between medication adherence and glycemic control.

## Results

Participants (*N* = 151) were on average age 55.3 ± 11 years, 60.9% female, 76.2% White, 90.1% had at least a high school education, and 74.2% had incomes >$40,000. Average years since a T2D diagnosis was 9.9 ± 7.3 years, and the average A1c was 8% ± 1.5%. Participants scored 12.1 ± 1.3 on the BHLS (sample range, 7–13), 4.5 ± 1 on the SNS (sample range, 1–6), and 81.5% had optimal medication adherence according to the ARMS-D (**Table 1**).

Compared to participants who were optimally adherent, participants who were suboptimally adherent had lower health literacy skills (*U* = 1,303, *p* = .027), lower numeracy skills (*U* = 1,200, *p* = .012), and worse glycemic control (*U* = 1,244.5, *p* = .043). Participants who were optimally and suboptimally adherent did not differ by age, gender, race, education, income, insurance status, insulin use, duration of diagnosed diabetes, or the number of prescribed diabetes medications.

In an unadjusted logistic regression model, health literacy skills were significantly associated with medication adherence. For every 1-point increase on the BHLS, participants were 1.6 times more likely to have optimal medication adherence (*p* < .05), 95% confidence interval (CI) [1 – 2.5]. In the adjusted model, for every 1-point increase on the BHLS, participants were 1.8 times more likely to have optimal medication adherence (*p* < .05), 95% CI [1.1 – 3.0].

In a second unadjusted logistic regression model, numeracy skills were significantly associated with medication adherence. For every 1-point increase on the SNS, participants were 1.9 times more likely to have optimal medication adherence (*p* < .05), 95% CI [1.1 – 3.1]. In the adjusted model, for every 1 point increase on the SNS, participants were 2.7 times more likely to have optimal medication adherence (*p* < .01), 95% CI [1.4 – 5.1].

## Discussion

We examined relationships between health literacy skills, numeracy skills, and medication adherence, and, separately, the relationship between medication adherence and glycemic control among a sample of people with T2D. Limited health literacy and limited numeracy skills were each associated with suboptimal medication adherence. Suboptimal medication adherence was, in turn, associated with having worse glycemic control.

Our findings suggest health literacy and numeracy skills have an important role in helping people regularly take their diabetes medication(s). When we controlled for factors associated with suboptimal medication adherence such as younger age (Kirkman et al., 2015), female gender (Kirkman et al., 2015), having a low income (Rolnick, Pawloski, Hedblom, Asche, & Bruzek, 2013), less education (Rolnick et al., 2013), health literacy and numeracy skills remained significant predictors of adherence. Simplifying instructions and using images rather than numbers can overcome literacy and numeracy limitations, and may foster better medication taking. However, additional strategies for communicating medication management and adherence information are needed (Cavanaugh et al., 2009).

Multiple measures are used to assess health literacy and numeracy skills (Kiechle, Bailey, Hedlund, Viera, & Sheridan, 2015). Health literacy assessed with the Rapid Estimate of Adult Literacy in Medicine (REALM) has been associated with medication adherence assessed with the Summary of Diabetes Self-Care Activities medications subscale (Osborn, Cavanaugh, et al., 2011). Diabetes-specific numeracy skills measured with Diabetes Numeracy Test (DNT) has also been associated with better glycemic control (Osborn, Cavanaugh, Wallston, White, & Rothman, 2009). Our findings build on the current literature with other valid and reliable measures of health literacy skills (i.e., BHLS), numeracy skills (i.e., SNS), and medication adherence (i.e., ARMS-D).

The BHLS and SNS are subjective measures of health literacy and numeracy skills, respectively, whereas the REALM and DNT are objective measures of health literacy and diabetes-specific numeracy skills, respectively. The BHLS and SNS correlate well with objective health literacy/numeracy measures, such as the Shortened Test of Functional Health Literacy in Adults and Wide Range Achievement Test 3 (McNaughton, Cavanaugh, Kripalani, Rothman, & Wallston, 2015; Wallston et al., 2014), but, unlike objective measures, are briefer and place less burden on participants (McNaughton, Wallston, Rothman, Marcovitz, & Storrow, 2011).

There are limitations to acknowledge. First, cross-sectional data can describe associations between variables, but cannot infer causation. Prospective research is needed to determine the effects of health literacy and numeracy skills on medication adherence over time, allowing for inferences about the directionality of these relationships. Additionally, we recruited our participants from a single academic center limiting the generalizability of our findings to other populations. Our sample was predominately White, college-educated, with higher incomes, and higher literacy and numeracy skills. Findings may not generalize to more racially/ethnically and socioeconomically diverse populations with the highest prevalence of diabetes in the United States, including people with lower literacy and numeracy skills.

Health literacy and numeracy skills should be considered in the design of education materials to promote medication management and adherence. Employing techniques such as the use of plain language and the Teach-Back method (Osborn, Cavanaugh, & Kripalani, 2010) helps all people, regardless of their literacy and numeracy skills. However, with many people using digital mediums to learn about and manage medications (e.g., order refills, request reauthorizations), the development of clear and effective electronic communications (e.g., infographics, simple directives) to promote medication taking are sorely needed.

## Figures and Tables

**Table 1:** Participant Characteristics

**Characteristic**	**Total (*N* = 151) (mean ± *SD* or *n* [%])**	**Medication Adherence**	

		**Suboptimal**	**Optimal**

		*n* = 28 (18.5)	*n* = 123 (81.5)

Age (years)	55.3 ± 11	55.1 ± 10.8	56.2 ± 12.1

Gender (female)	92 (60.9)	75 (61)	17 (60.7)

Race			
White	115 (76.2)	94 (76.4)	21 (75)
Non-White	36 (23.8)	29 (23.6)	7 (25)

Education			
None or kindergarten	0 (0)	0 (0)	0 (0)
Grades 1–5	0 (0)	0 (0)	0 (0)
Grades 6–8	1 (.7)	0 (0)	1 (.8)
Grades 9–11	14 (9.3)	5 (17.9)	9 (7.3)
Grade 12 (or GED)	56 (37.1)	10 (35.7)	46 (37.4)
Some college	51 (33.8)	6 (21.4)	45 (36.6)
Graduated college	29 (19.2)	7 (25)	22 (17.9)
Graduate school	0 (0)	0 (0)	0 (0)

Income			
≤$40,000	39 (25.8)	30 (24.4)	9 (32.1)
>$40,000	112 (74.2)	93 (75.6)	19 (67.9)

Insurance status^a^			
Private	127 (84.1)	104 (84.6)	23 (82.1)
TennCare/Medicare	23 (15.2)	18 (14.6)	5 (17.9)

Diabetes			
Insulin use	68 (45)	58 (47.2)	10 (35.7)
Years of T2D diagnosis	9.9 ± 7.3	10.3 ± 7.2	8.3 ± 7.8
Number of T2D medications	2 ± 0.9	2 ± 0.9	1.8 ± 0.8

Numeracy (SNS), range, 1–6	4.5 ± 1	4.4 ± 1.1	4.9 ± 0.8

Health literacy (BHLS), range, 3–15	12.1 ± 1.3	12 ± 1.4	12.5 ± 0.9

Medication adherence (ARMS-D), range, 11–44	39.6 ± 4.2	38.6 ± 4	44 ± 0

Glycemic control (hemoglobin A1c%)	8 ± 1.5	8.1 ± 1.6	7.4 ± 1.1

Note. ARMS-D = Adherence to Refills and Medications Scale for Diabetes; BHLS = Brief Health Literacy Screen; GED = general equivalency diploma; SNS = Subjective Numeracy Scale; T2D = type 2 diabetes.

a *n* = 1 missing data.
